# Stunting in infancy, pubertal trajectories and adult body composition: the Birth to Twenty Plus cohort, South Africa

**DOI:** 10.1038/s41430-020-00716-1

**Published:** 2020-08-14

**Authors:** Glory Chidumwa, Rihlat Said-Mohamed, Lukhanyo H. Nyati, Feziwe Mpondo, Tinashe Chikowore, Alessandra Prioreschi, Juliana Kagura, Lisa J. Ware, Lisa K. Micklesfield, Shane A. Norris

**Affiliations:** 1 SAMRC/Wits Developmental Pathways for Health Research Unit, Department of Paediatrics and Child Health, School of Clinical Medicine, Faculty of Health Sciences, University of the Witwatersrand, 7 York Rd, Parktown, Johannesburg, 2193 South Africa; 2 Department of Archaeology, Faculty of Human, Social and Political Science, School of Humanities and Social Sciences, University of Cambridge, Cambridge, UK; 3 Division of Epidemiology and Biostatistics, School of Public Health, University of the Witwatersrand, Johannesburg, South Africa; 4 Institute of Developmental Sciences, University of Southampton, Southampton, UK

**Keywords:** Epidemiology, Paediatrics

## Abstract

**Background/objectives:**

Childhood rapid growth and earlier puberty onset have been associated with adult obesity. However, the association between childhood stunting, pubertal timing and adult obesity is unclear. We examined whether the relationship between stunting at age 2 years (y) and body composition at 23 years is mediated by adolescent body mass index, and pubertal development, using the Birth-to-Twenty Plus cohort (South Africa).

**Subjects/methods:**

For 1036 participants, data on anthropometrics between birth and 23 years, maternal factors, and pubertal development (Tanner scale at 9–16 years) were collected. Stunting at 2 years (height-for-age *z*-score < −2), 5–18 years BMI-for-age trajectories, pubertal development trajectories, and DXA-derived fat mass (FM) and fat free mass (FFM) at 23 years were determined. Data were analysed using hierarchical regressions and structural equation models.

**Results:**

Stunting was directly associated with slower pubertal development and with shorter adult stature, but was not associated with adolescent BMI trajectories, adult FM or FFM. However, stunting was indirectly associated with adult FM and FFM through the direct associations between slower pubertal development and lower FM and between shorter height and lower FFM. BMI trajectories were independently associated with FM and FFM.

**Conclusions:**

Being stunted in this population predicted adult body composition through slower pubertal development and shorter adult stature.

## Introduction

In low- and middle-income countries (LMICs), obesity has become a critical determinant of morbidity and mortality due to its association with higher risks of cardiovascular and metabolic diseases [1]. The rapid nutrition transition, which intersects with persistent undernutrition in LMICs poses a double burden of malnutrition [2]. In 2016, 44% of overweight children under 5 years of age and 66% of stunted children under 5 years of age were reported to be living in LMICs [3]. While there has been a decrease in the prevalence of stunting globally, the trends in Africa are worrisome. It is the only region that has experienced an increase in the prevalence of stunting in children under 5 years of age while the number of overweight children of the same age has increased by nearly 50% since the year 2000, and 12% of adults are also obese [3, 4].

A life-course approach may contribute to a better understanding of the antecedents of obesity. Physical growth during the developmental period from conception through to early childhood may be a critical determinant of adult body composition and obesity risk [5]. In LMICs, both rapid linear growth and weight gain between birth and 2 years of age have been shown to contribute to greater adult lean mass and height in comparison to adult fat mass (FM), while from 2 years of age onwards evidence is consistent with observations in high income countries, which shows that rapid weight gain becomes a risk factor for overweight/obesity, and greater FM [6–11]. During the adolescent transition, pubertal timing (onset) and progression (tempo, speed of pubertal development) may also play an important role in influencing body composition and obesity risk in adulthood [12, 13]. Early and late onset of menarche and faster progression through puberty have been associated with higher body mass index (BMI) and adiposity levels in adulthood [12]. Findings from LMICs have consistently shown that greater height and weight gain during early childhood predict an earlier onset of puberty and a faster tempo of pubertal development [14, 15]. In addition, both greater growth rate during childhood and faster pubertal development during adolescence were found to be risk factors for overweight and obesity in young adulthood [6, 10]. Findings from LMICs with regards to whether stunting plays a role are less clear. Childhood stunting has been associated with late onset pubertal development [16, 17]. However, stunting in children has not been consistently associated with the risk of overweight or obesity in childhood and adolescence [18**–**22].

South Africa is experiencing a double burden of malnutrition; it has one of the highest prevalence of obesity in adults and children in the sub-Saharan region, while 26% of children under 3 years of age are classified as stunted [23]. The South African Birth-to-Twenty Plus prospective cohort study (BT20+) is the largest and longest running (1990–present) birth cohort in sub-Saharan Africa, and offers a unique opportunity to explore the associations between undernutrition in childhood, pubertal development, and risks of obesity in adulthood [24]. In previous analyses of data from the BT20+ study [22, 25], stunting at 2 years of age was not associated with BMI nor with overweight and obesity risks between 4 and 24 years age. However, using imaging data (DXA) of the BT20+ participants, results consistently showed that stunting at 2 years of age was associated with lower fat free mass (FFM) at 7–9 years, 10 years and 23 years of age [8, 25, 26]. In addition, in the same cohort, males’ and females’ pre-pubertal weight and height as well as the timing of the onset of pubertal development were associated with BMI in young adulthood suggesting that pubertal development may mediate the associations between growth in childhood and body composition in young adulthood [13]. Using hierarchical regressions and structural equation modelling (SEM), this study aimed to determine whether changes in BMI from childhood through to late adolescence, and/or the tempo of pubertal development, mediate the association between stunting, and height and body composition (FM and FFM) in young adulthood in the BT20+ cohort.

## Methods

### Study design and setting

The Birth-to-Twenty Plus prospective cohort was set up to observe growth, development and health in an urban cohort following the democratic transition in the Republic of South Africa. Inclusion and exclusion criteria for the BT20+ study have been described elsewhere [24]. Between 23 April and 8 June 1990, this birth cohort study included 3273 mothers and their singleton newborns born in Soweto (Johannesburg, South Africa) who were to remain in the area until the child turned 6 months of age. Since their birth, information on growth, diet, physiological indicators, and health have been collected amongst other characteristics at each of the 23 data collection waves, and the current wave at age 28 years has just been completed. The Human Research Ethics Committee of University of the Witwatersrand (South Africa) granted ethical clearance for this study (M111182) and the study was conducted in line with the Principles of the Declaration of Helsinki for research involving human subjects. Participants and their caregivers provided written informed assent (where appropriate) and consent.

### Anthropometric indices and trajectories

Anthropometric data were collected regularly from birth to 23 years. Weight and supine length up to age 2 years and standing height after age 2 years, were collected by trained research assistants using standard techniques [27]. For each participant, height to the nearest 0.1 cm was measured using a wall-mounted stadiometer (Holtain, UK) and weight to the nearest 0.1 kg was measured using a digital scale with participants in light clothes and without shoes.

#### Stunting at age 2 years

Height-for-age *z*-score (HAZ) at age 2 years was derived from the World Health Organization sex-specific growth standards [28] and stunting was defined as HAZ < −2.

#### BMI trajectories between 5 years and 18 years

BMI trajectory groups from five years to 18 years have been previously determined using latent class growth mixture modelling (Supplementary Fig. S1) [29]. The characterisation of BMI trajectories permitted the identification of sex-specific adiposity patterns and tracking from early childhood to late adolescence. Females could belong to one of the following four BMI trajectory groups: “(1) normal weight (2), late onset overweight (3), early onset obesity to overweight and (4) early onset obesity to morbid obese”. Male trajectory groups were identified and classified as: “(1) normal weight (2), early onset overweight to normal and (3) early onset overweight to obese”.

#### Pubertal development trajectories

The Tanner sexual maturation scale was used to determine the stage of pubertal development from 9 to 17 years. Sex-specific pubertal development trajectory classes have been determined previously using latent class growth analyses [14]. Four classes of breast development were identified for girls and four classes of genital development were identified for boys (Supplementary Fig. S2). For boys’ and girls’ trajectories, participants in trajectory class 1 had the slowest pubertal development, while those in the highest trajectory class were characterised by the fastest pubertal development.

#### Fat mass and fat free mass at age 23 years

Participants’ whole body composition was measured according to standard procedures with dual-energy x-ray absorptiometry (DXA; Hologic QDR 4500A, Bedford, USA) by a trained technician. A daily calibration of the DXA machine was performed using a phantom spine; coefficients of variation for total FM and FFM were <2% and 1%, respectively. Each scan was analysed excluding the head with the DXA software version 4.0.2 (Hologic Inc., Bedford, USA) to derive whole body FM and FFM.

### Maternal factors

Interviewer-administered questionnaires were completed with the mothers or caregivers of the BT20+ participants when participants were 6 months, 1 year and 2 years of age. Information on maternal education, parity, and age was collected at each occasion. A household socio-economic index was calculated by summing all the physical/economic assets owned in the household [30].

### Study sample

Participants included in this study were all BT20+ participants at 23 years of age who had not been pregnant between 17 and 23 years of age, and had the following data: anthropometry at 2 years of age, pubertal trajectories from 9 to 17 years, BMI trajectories between 5 years and 18 years, and DXA-derived body FM and FFM at 23 years of age.

### Statistical analyses

**Fig. 1:**
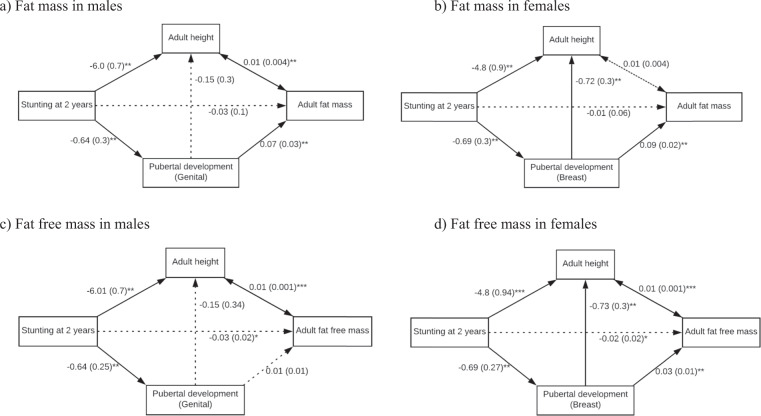
Structural equation models for the association between stunting and fat mass and fat free mass in male and female participants of the Birth-to-twenty Plus cohort study. The solid lines represent significant direct effects and the dashed line is not a significant direct effect. All values are coefficients with standard errors in parenthesis. Significance levels: *p value < 0.1; **p value < 0.05; ***p value < 0.001. The model fit statistics were: the Root Mean Square Error of Approximation for (**a**): 0.022 (0.019–0.024), (**b**): 0.027 (0.023–0.32), (**c**): 0.42 (0.39–0.44), (**d**): 0.016 (0.009–0.021); the Comparative Fit Index for (**a**): 0.781, (**b**): 0.920, (**c**): 0.844, (**d**): 0.764; the Tucker–Lewis Index for (**a**): 0.778, (**b**): 0.905, (**c**): 0.800, (**d**): 0.759.

Stata software 15.1 (StataCorp, College Station, TX: Stata Corporation) was used for all analyses. Normality was tested using the Shapiro–Wilk test, and study characteristics were compared between males and females using the Wilcoxon sum-rank and chi-squared tests. In males and females, bivariate analyses were performed to assess the associations between maternal factors, stunting at age 2 years, and pubertal development and body composition, using linear regression and multinomial logit models. In addition, hierarchical regressions were performed to assess the associations between stunting and adult FM and FFM, and to assess the potential contributions of pubertal development in the adolescent period and adult height. Model 1 was unadjusted, in Model 2 adolescent pubertal trajectories was controlled for, and in Model 3 adolescent pubertal trajectories and adult height were controlled for. Then, SEM analyses were used to determine whether pubertal development and adult height mediated the association between stunting at 2 years and adult body composition (FM and FFM). Multivariable analyses and SEM were based and guided by an a priori conceptual model (Fig. 1). Bold lines represent statistically significant paths while dotted lines represent paths that were not statistically significant. We conducted analyses separately for males and females.

## Results

### Descriptive statistics

A total of 1036 participants were included in the analyses (Table 1). At age 2 years, 27.4% of males and 18.4% of females were stunted (Table 1). Through 9–16 years of age, 25% of females were in the fastest pubertal development trajectory compared to 11% of the males who were in the fastest trajectory. The majority of participants (94% of males and 83% of females) followed the “*normal weight”* BMI trajectory. At age 23 years, males and females differed in their stature and body composition; females were shorter by 11.9 cm (*p* < 0.001), had a higher BMI (24.3 vs. 20.8 kg.m^2^, *p* < 0.001), had twice as much FM as males (*p* < 0.001), and 25% less FFM (*p* < 0.001).

**Table 1:** Developmental characteristics of the Birth-to-twenty Plus cohort participants through age 23 years and maternal characteristics.

Variables	All (*N*)	Males	Females	*p* values
BT20+ characteristics				
*Childhood through late adolescence*				
Stunting at age 2 years^a^				
*No*	458	209 (72.6)	249 (81.6)	0.008
*Yes*	135	79 (27.4)	56 (18.4)	
Pubertal development trajectories				
1 (slowest)	222	126 (24.3)	96 (18.8)	N/A
2	331	197 (38.0)	134 (26.3)	
3	289	136 (26.3)	153 (30.0)	
4 (fastest)	186	59 (11.4)	127 (24.9)	
BMI trajectories^b^ (*N* = 810)				
0	804	436 (94.0)	368 (82.5)	N/A
1	68	23 (5.0)	45 (10.1)	
2	19	5 (1.1)	14 (3.1)	
3	19	N/A	19 (4.3)	
*At age 23 years*				
Age (years)^c^	996	23.3 (22.5; 23.5)	23.2 (22.6; 23.5)	0.57
Height (cm)^c^	997	171.8 (167.5; 175.7)	159.9 (155.2; 164.0)	<0.001
Weight (kg)^c^	989	61.4 (55.8; 69.9)	61.8 (53.6; 71.6)	0.83
Body mass index. (kg/m^2^)^c^	989	20.8 (19.2; 23.3)	24.32 (21.0; 28.5)	<0.001
Fat Mass (kg)^c^	990	11.0 (8.7; 15.1)	22.8 (17.4; 30.4)	<0.001
Fat free mass (kg)^c^	1036	43.8 (39.9; 48.0)	32.6 (30.0; 36.6)	<0.001
*Maternal characteristics*				
Maternal Parity^a^				
*One child*	382	195 (37.3)	187 (36.5)	0.78
*More than one child*	654	328 (62.7)	326 (63.6)	
Maternal Education^a^				
*Primary*	125	71 (13.6)	54 (10.5)	0.02
*Secondary*	437	234 (44.7)	203 (39.6)	
*University/college*	474	218 (41.7)	256 (49.9)	
Maternal age at birth (years)	1035	25 (21; 30)	25 (21; 30)	0.98

*N/A* not applicable.

^a^Values are N (Frequencies).

^b^Males BMI trajectories: (0) normal weight, (1) early onset overweight to normal and (2) early onset overweight to obese; Females BMI trajectories: (0) normal weight (1), late onset overweight (2), early onset obesity to overweight and (3) early onset obesity to morbid obese.

^c^Values are Medians (Inter-Quartile range (IQR)).

### Bivariate analyses

In bivariate analyses (Table 2), stunting at 2 years was not associated with adult FM or FFM in males or females. In males, stunting was associated with a lower relative risk of being in faster pubertal trajectories 2, 3 and 4, and a lower adult height by 6.1 cm (*p* < 0.001). In females, stunting was associated with a lower relative risk of being in the faster pubertal trajectories 3 and 4, and with lower adult height by 4.5 cm (*p* < 0.001). Stunting was not associated with BMI trajectories in males, and in females it was associated with a higher relative risk to have a later onset overweight trajectory 1. In both sexes BMI trajectories had stronger associations with FM and FFM than pubertal trajectories (Table 3). Both pubertal and BMI trajectories had stronger associations with FM compared to FFM (Table 3).

**Table 2:** Bivariate associations between stunting at age 2 years and pubertal development, adult height and body composition in male and female participants of the Birth-to-twenty Plus cohort study (*N* = 1036).

Variables	Males	Females
	RRR (95% CI)	RRR (95% CI)
*Pubertal development trajectories*		
1 vs. 2	0.48 (0.24; 0.95)**	1.18 (0.55; 2.54)
1 vs. 3	0.45 (0.22; 0.92)**	0.41 (0.17; 1.00)*
1 vs. 4	0.31 (0.12; 0.80)**	0.45 (0.17; 1.15)*
*BMI trajectories*		
0 vs. 1	1.02 (0.26; 3.95)	3.58 (1.58; 8.15)**
0 vs. 2	1.35 (0.12; 15.21)	–
0 vs. 3		0.98 (0.21; 4.62)
	Coefficient (95% CI)	Coefficient (95% CI)
Height at 23 years	−6.1 (0.73)***	−4.5 (0.93)***
Fat mass at 23 years	−0.04 (0.06)	−0.03 (0.06)
Fat free mass at 23 years	−0.33 (0.25)	−0.37 (0.28)

Analyses are: multinomial logit models (risk ratios and 95% confidence intervals) for pubertal development and BMI trajectories; linear regression models (coefficient are simple linear regression coefficients; standard errors in parenthesis) for adult height, fat mass and fat free mass. Significance levels: *p < 0.05; **p < 0.01 ***p < 0.001; – shows perfect prediction and hence no estimates. All estimates are for stunted participants, with non-stunted participants as the reference. Males BMI trajectories: (0) normal weight, (1) early onset overweight to normal and (2) early onset overweight to obese; Females BMI trajectories: (0) normal weight, (1) late onset overweight (2), early onset obesity to overweight and (3) early onset obesity to morbid obese.

**Table 3:** Bivariate associations between pubertal development trajectories, BMI trajectories and adult height, and adult body composition, in male and female participants of the Birth-to-twenty Plus cohort study.

Variables	Males		Females	
	*N*	Coefficient (SE)	*N*	Coefficient (SE)
Fat mass				
*Pubertal development trajectories*				
1 - Slowest	123	Reference	90	Reference
2	187	0.07 (0.05)	129	0.11 (0.05)*
3	129	0.17 (0.05)**	144	0.08 (0.05)
4 - Fastest	58	0.15 (0.07)*	122	0.27 (0.05)**
*BMI trajectories*				
0	419	Reference	350	Reference
1	22	0.59 (0.08)**	44	0.28 (0.06)**
2	5	0.86 (0.17)**	13	0.28 (0.10)**
3		N/A	18	0.64 (0.09)**
Height at 23 years	482	0.01 (0.003)**	470	0.01 (0.003)
Fat free mass				
*Pubertal development trajectories*				
1 - Slowest	126	Reference	96	Reference
2	197	0.02 (0.02)	134	0.02 (0.02)
3	136	0.03 (0.02)*	153	0.02 (0.02)
4 - Fastest	59	0.04 (0.02)	127	0.09 (0.02)**
*BMI trajectories*				
0	436	Reference	368	Reference
1	23	0.07 (0.03)*	45	0.05 (0.02)*
2	5	0.24 (0.06)**	14	0.10 (0.04)**
3		N/A	19	0All values are linear regression coefficients with standard errors in parentheses; Significance.20 (0.03)**
Height at 23 years	503	0.01 (0.003)**	494	0.01 (0.003)**

All values are linear regression coefficients with standard errors in parentheses; Significance levels: **p < 0.01, *p < 0.05. Males BMI trajectories: (0) normal weight, (1) early onset overweight to normal and (2) early onset overweight to obese; Females BMI trajectories: (0) normal weight, (1) late onset overweight (2), early onset obesity to overweight and (3) early onset obesity to morbid obese.

### Hierarchical regression

Stunting at 2 years was not associated with adult FM in males or females (Table 4). In males, pubertal development was associated with FM only when height was adjusted for (Model 3) and an increase in adult height was significantly associated with greater FM. In females, participants within the faster pubertal development trajectory class had significantly higher FM compared to their counterparts within the slowest pubertal development trajectory class. Contrasting with males, females’ adult height was not associated with FM.

**Table 4:** Unadjusted and adjusted associations between stunting at 2 years and fat mass at 23 years in male and female participants of the Birth-to-twenty Plus cohort study.

Variables	Males						Females					
	*N*	Model 1	*N*	Model 2	*N*	Model 3	*N*	Model 1	*N*	Model 2	*N*	Model 3
*Stunting at 2 years*												
No	198	Reference	197	Reference	188	Reference	238	Reference	238	Reference	230	Reference
Yes	79	−0.10 (0.06)	77	−0.10 (0.06)	75	−0.03 (0.07)	54	−0.08 (0.06)	53	−0.04 (0.06)	50	−0.01 (0.06)
*Pubertal development trajectories*												
1 - Slowest			59	Reference	57	Reference			55	Reference	54	Reference
2			95	0.05 (0.07)	89	0.10 (0.07)			85	0.05 (0.07)	82	0.06 (0.07)
3			80	0.11 (0.08)	78	0.13 (0.07)			84	0.09 (0.07)	80	0.10 (0.07)
4 - Fastest			40	0.15 (0.09)	39	0.22 (0.09)*			67	0.28 (0.07)**	64	0.28 (0.07)**
Height at 23 years					263	0.01 (0.003)*					280	0.01(0.003)
Observations		277		274		263		292		291		280
R-squared		0.01		0.03		0.06		0.01		0.07		0.07

All values are linear regression coefficients with standard errors in parentheses; Significance levels: *p < 0.05; **p < 0.01.

Concerning FFM (Table 5), stunting was associated with lower FFM in males and females at 23 years before adjusting for adult height. An increase in adult height was then significantly associated with greater adult FFM in both sex. For both sexes, in model 1 and 2 stunting was associated with a reduced FFM of ~90 g. In model 3, adjusting for height, this association was no longer significant, but an increase by 1 cm in adult height was associated with greater FFM by 10 g. These results suggest that adult height may mediate the association between stunting at 2 years of age and FFM. In males, while the addition of pubertal development in Model 2 did not change the variance explained by the model, the addition of adult height in Model 3 explained an additional 16% of the variance in adult FFM. In females, the addition of pubertal development in Model 2 explained an additional 3% of the variance in adult FFM, while the addition of adult height in Model 3 explained an additional 20% of the variance in adult FFM.

**Table 5:** Unadjusted and adjusted associations between stunting at 2 years and fat free mass at 23 years in male and female participants of the Birth-to-twenty Plus cohort study.

Variables	Males						Females					
	*N*	Model 1	*N*	Model 2	*N*	Model 3	*N*	Model 1	*N*	Model 2	*N*	Model 3
*Stunting at 2 years*												
No	209	Reference	208	Reference	199	Reference	249	Reference	249	Reference	240	Reference
Yes	79	−0.09 (0.02)**	77	−0.09 (0.02)**	75	−0.03 (0.02)	56	−0.09 (0.02)**	55	−0.08 (0.02)**	52	−0.02(0.02)
*Pubertal development trajectories*												
1 - Slowest			61	Reference	59	Reference			60	Reference	59	Reference
2			100	0.02 (0.02)	94	0.03 (0.02)			87	0.00 (0.03)	84	0.01 (0.02)
3			84	0.01 (0.02)	82	0.01 (0.02)			90	0.00 (0.02)	85	0.03 (0.02)
4 - Fastest			40	0.03 (0.03)	39	0.05 (0.03)*			67	0.07 (0.03)**	64	0.09 (0.02)**
Height at 23 years					274	0.01 (0.003)**					292	0.01 (0.003)**
Observations		288		285		274		305		304		292
R-squared		0.09		0.09		0.25		0.05		0.08		0.28

All values are linear regression coefficients with standard errors in parentheses; Significance levels: *p < 0.05; **p < 0.01.

### Structural equation modelling

In both males and females, stunting at 2 years had no direct effect on FM or FFM. In males, stunting had statistically significant indirect (through adult height and through pubertal trajectory) and total effect on FM (Fig. 1a). These results were similar in females; however, stunting had an indirect effect on FM only through pubertal trajectory (Fig. 1b).

For FFM, the total effect of stunting in males and females was statistically significant. Furthermore, for both sexes, being stunted was indirectly associated with FFM through adult height. Stunting at 2 years was indirectly associated with FFM through pubertal trajectory in females but not males (Fig. 1c, d).

## Discussion

We demonstrated that the association between stunting at 2 years of age and adult body composition was moderated by attained adult height and by the tempo of pubertal development. In this population, we show that males and females who were previously stunted were more likely to have a slower pubertal development and a shorter adult height. In stunted males, slower pubertal development and shorter adult height predicted lesser adult FM, while lower FFM was only predicted by shorter adult height. In females, slower pubertal development predicted lower FM, while slower pubertal development and shorter adult stature predicted lower FFM.

The thrifty phenotype hypothesis suggests that children with impaired growth during intrauterine and infancy periods are at greater risk of developing type 2 diabetes and the metabolic syndrome [31]. These disease may be induced by changes in glucose and insulin metabolisms as well as physiological energy sparing mechanisms resulting from the exposure to undernutrition in early life [31]. Building on this hypothesis, it has been suggested that in response to early-life undernutrition, stunted children may be at a greater risk of storing fat, and of becoming overweight or obese adults, leading to the double burden of malnutrition at individual level [32, 33]. Impaired lipid metabolism [32], reduced resting energy expenditure [32, 34, 35], decreased physical activity levels [34, 35], and/or opportunistic overeating behaviour [36] are some of the mechanisms proposed by which stunted children may accumulate FM. Cross-sectional surveys have found contrasting results regarding the risk for stunted children to develop overweight and obesity during childhood or adolescence; either an increased risk or no association [18, 37, 38]. In a cross-sectional sample of 10–15 year old adolescents in South Africa there was no association between stunting and overweight [18], while a study using data from a cross-sectional national survey found that stunted children under the age of 6 years had a relative risk of 2.6 (2.0–3.4) of being overweight [37]. Our results support and expand findings from longitudinal studies, which have consistently reported no direct association between stunting in early life and the risk of overweight or obesity during childhood or adolescence [21, 22, 25]. Indeed, using hierarchical regressions and then SEM, our study shows an indirect association between stunting during childhood and adult body composition. Our data suggests that previously stunted persons are more likely to become shorter adults [39, 40] who, in our population, are in turn characterised by lower FM and FFM in males, and lower FFM in females. Stunting has been associated with lower FM and FFM in childhood, adolescence and adulthood [25, 41, 42]. A shorter adult stature in previously stunted children may translate into lower bone and muscle mass, resulting in less FFM in comparison to their non-stunted counterparts [8, 34]. In cases of continued undernutrition, stunted children may preferentially use energy from protein stored in muscles with such catabolism also potentially resulting in reduced muscle mass [43]. Our results are consistent with large cohort studies, which have found that in LMICs rapid length and weight gain in the first 2 years of life predicted greater adult height and lean mass [9, 10, 33]. Concerning adiposity, stunting has been associated with greater central and visceral FM during adolescence and adulthood (as measured by DXA, ultrasound and other techniques) suggesting that stunting may have a stronger effect on body fat distribution rather than on whole body FM [44].

Previous studies in LMICs have found that stunting was associated with later onset of puberty [16, 17, 45]. Our data extends this by showing that stunting was associated with slower pubertal development and that, in turn, slower pubertal development predicted lower FM in adult males and females, and lower FFM only in females. Sex differences in these associations during the pubertal transition have also been reported in Brazil [46]. The adolescence transition is characterised by significant changes in body composition associated with pubertal development [47]. In this study, BMI trajectories through childhood and adolescence had the strongest associations with adult FM. Our results support this for both sexes, with transient (temporary periods of) overweight or obesity during adolescence independently conferring greater body FM and FFM in adulthood [6, 12, 13, 48]. During pubertal development, both males and females gain in adiposity, but this is more pronounced in females [47]. In males, a marked increase in muscle mass is expected [47]. In the present study, in both sexes, a faster pubertal development was associated with greater adult FM and FFM, with stronger associations between faster pubertal development and FM. This finding adds to our understanding of the respective associations between the onset of and the tempo of menarche and adiposity levels [12]. Observed differences between males and females may also be related to hormonal and behavioural changes during the adolescence transition [49, 50].

The strength of this study is in the longitudinal assessment of the role of adolescent BMI trajectories and pubertal development in the association between stunting with adult body composition. The results from the present study adds to previous finding from this cohort by showing that the effect of early childhood stunting on FFM is on the decrease in adult height and the slow-down of pubertal tempo [8, 22, 25, 26]. However, this study is not without limitations; we have not investigated the association between stunting and FM distribution taking into account adolescent BMI and pubertal development; specific analyses will explore these particular associations using the combination of body composition data measured by DXA and ultrasound. While warranted, these analyses will need to be treated with caution as DXA accuracy is limited in determining adiposity in obese participants, with poorer precision for regional distribution, particularly FM percentage at the trunk in comparison to total body FM [51, 52]. In addition, we have not included other environmental factors such as dietary patterns, physical activity or socio-economic status that may affect body composition. The effects of such factors accumulate throughout the life-course and are embodied in the observed body composition in adulthood [53, 54].

In conclusion, in this study stunting is associated with lower FM and FFM in young adults, acting through shorter adult height and slower pubertal development. The combination of shorter stature and lesser lean mass in LMICs, such as South Africa, where the prevalence of obesity is dramatically increasing, may lay the foundation for higher risks for cardio-metabolic diseases later in life. This hypothesis will be explored in the next follow-up of the BT20+. Our finding support that linear growth, weight gain, and changes in body composition should be monitored from childhood through adolescence to adulthood. There is an urgent need for life-course interventions starting from foetal period to promote trajectories that lead to healthier adult body composition.

## Supplementary information

Supplementary Figures
