# Supplementary material for: Stunting in infancy, pubertal trajectories and adult body composition: the Birth to Twenty Plus cohort, South Africa
Source: Eur J Clin Nutr. 2020 Aug 14;75(1):189–97. doi: 10.1038/s41430-020-00716-1 (PMC7817521; doi:10.1038/s41430-020-00716-1)

Supplementary figures

Stunting in infancy, pubertal trajectories and adult body composition: Birth to Twenty Plus cohort, South Africa

Figure S1

a) BMI trajectories in girls. BMI latent classes plotted together with Extended International Obesity Task Force (IOTF) Cut-Offs for BMI in girls.

b) BMI trajectories in girls. BMI latent classes plotted together with Extended International Obesity Task Force (IOTF) Cut-Offs for BMI in girls. (Original source: Munthali et al., 2016)

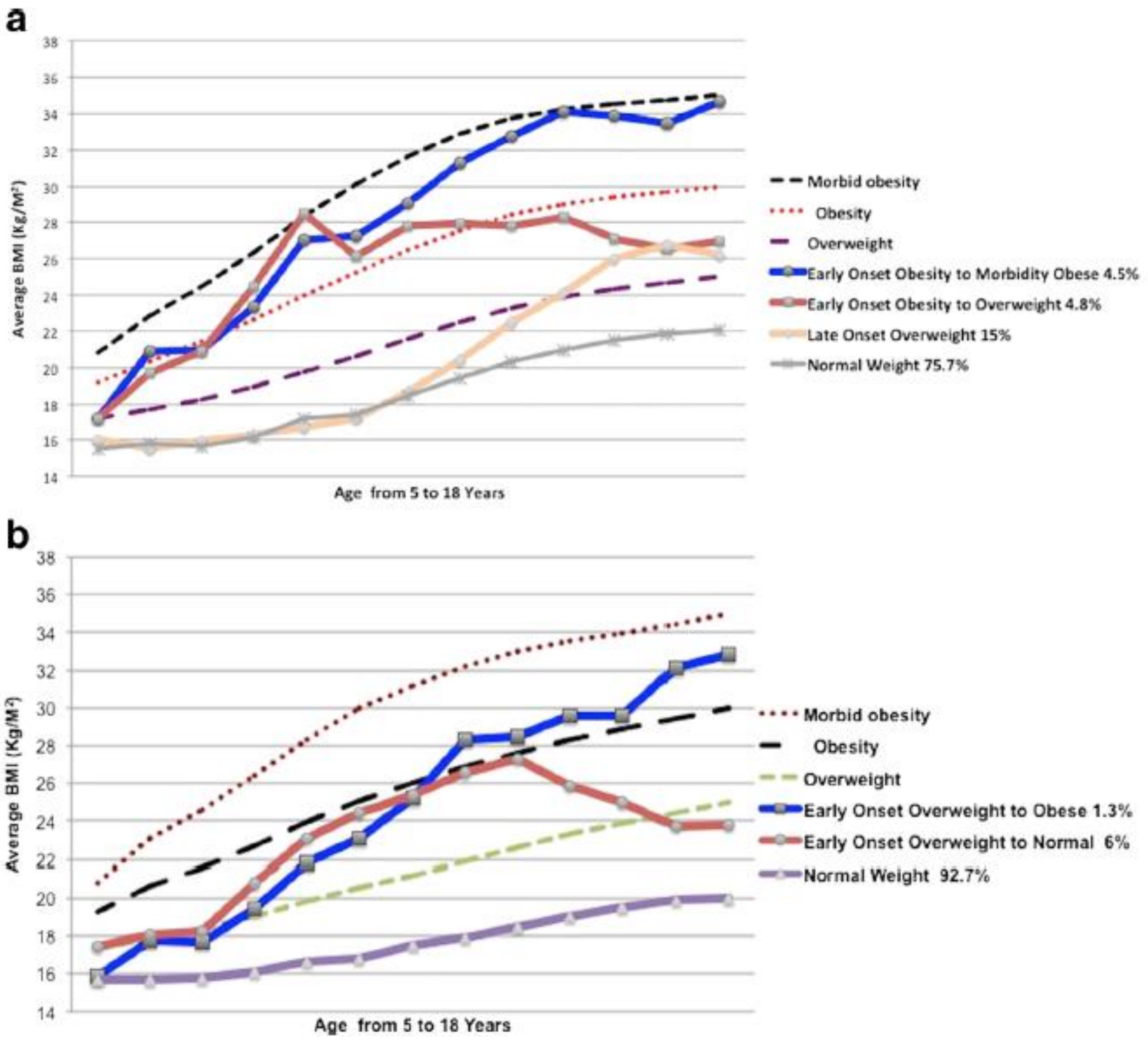

Figure S2
Mean Tanner scores for female (n=1135) and male (n=1060) pubertal development from 9 to 16 y of age by growth trajectory class and age, Bt20 study. Pubic hair, girls (A); breasts, girls (B); pubic hair, boys (C); and genitals, boys (D). (Original source: Lundeen et al., 2016)

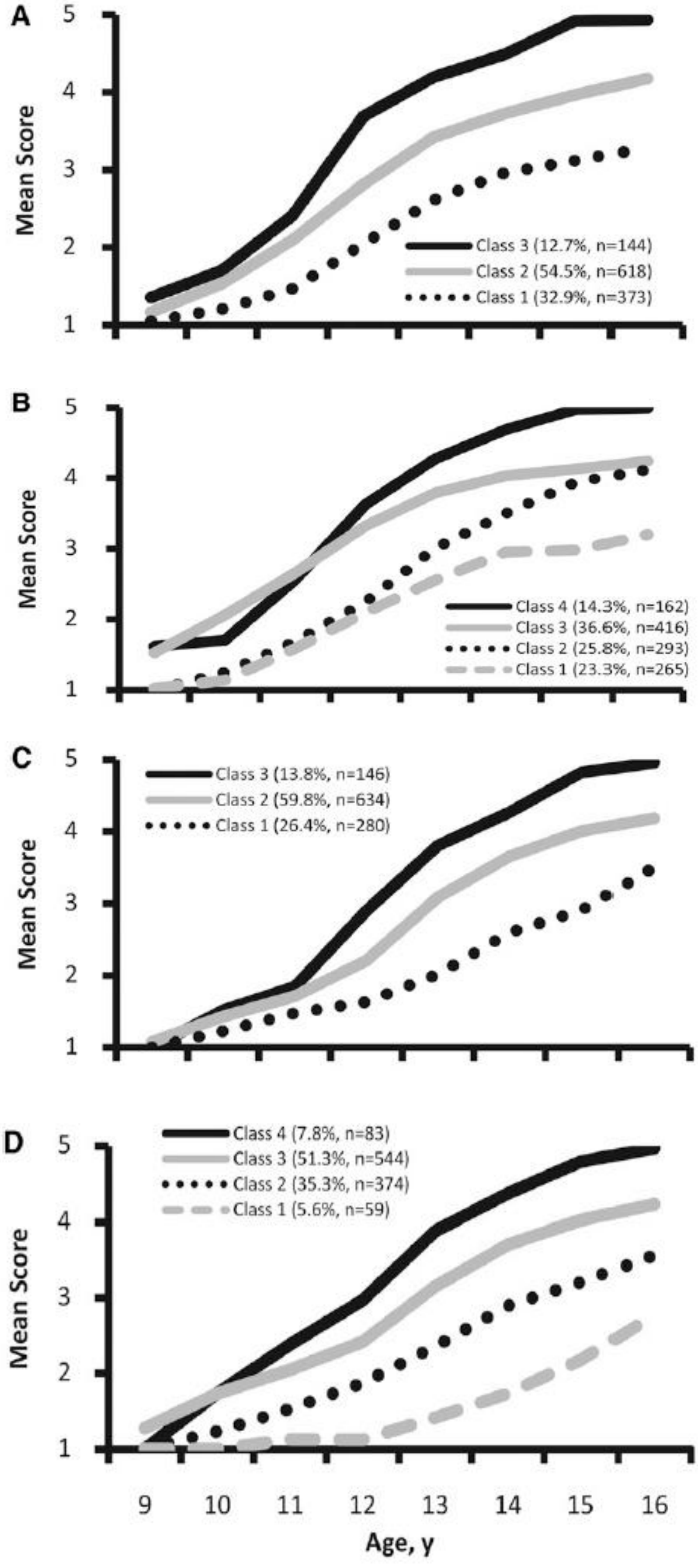

Supplement: Supplementary file 1 — Supplementary Figures [file 41430_2020_716_MOESM1_ESM.pdf]
